# Multi-Functional Sensing for Swarm Robots Using Time Sequence Classification: HoverBot, an Example

**DOI:** 10.3389/frobt.2018.00055

**Published:** 2018-05-17

**Authors:** Markus P. Nemitz, Ryan J. Marcotte, Mohammed E. Sayed, Gonzalo Ferrer, Alfred O. Hero, Edwin Olson, Adam A. Stokes

**Affiliations:** ^1^School of Engineering, Institute for Integrated Micro and Nano Systems, The University of Edinburgh, Edinburgh, United Kingdom; ^2^Department of Electrical Engineering and Computer Science, University of Michigan, Ann Arbor, MI, United States

**Keywords:** HoverBot, swarm robotics, multi-functional sensing, dynamic time warping, DTW, barycentre averaging, DBA

## Abstract

Scaling up robot swarms to collectives of hundreds or even thousands without sacrificing sensing, processing, and locomotion capabilities is a challenging problem. Low-cost robots are potentially scalable, but the majority of existing systems have limited capabilities, and these limitations substantially constrain the type of experiments that could be performed by robotics researchers. Instead of adding functionality by adding more components and therefore increasing the cost, we demonstrate how low-cost hardware can be used beyond its standard functionality. We systematically review 15 swarm robotic systems and analyse their sensing capabilities by applying a general sensor model from the sensing and measurement community. This work is based on the HoverBot system. A HoverBot is a levitating circuit board that manoeuvres by pulling itself towards magnetic anchors that are embedded into the robot arena. We show that HoverBot’s magnetic field readouts from its Hall-effect sensor can be associated to successful movement, robot rotation and collision measurands. We build a time series classifier based on these magnetic field readouts. We modify and apply signal processing techniques to enable the online classification of the time-variant magnetic field measurements on HoverBot’s low-cost microcontroller. We enabled HoverBot with successful movement, rotation, and collision sensing capabilities by utilising its single Hall-effect sensor. We discuss how our classification method could be applied to other sensors to increase a robot’s functionality while retaining its cost.

## 1. Introduction

### 1.1. Swarm Robotics

Swarm robotics is the study of developing and controlling large groups of simple robots. One goal of swarm robotics research is to substitute a few sophisticated robots with many simple robots to gain robustness, flexibility and to circumvent single-robot-failures from resulting in mission abortions (Brambilla et al., 2013). Applications range from space-exploration to finding survivors after large-scale disasters. Much inspiration in this area has been drawn from nature (Bonabeau et al., 1999). Flocks of birds fly in formation and take turns in positioning to maximise the total travelled distance as a collective. Schools of fish cluster together to increase the chances of survival against a visually orientated predator. Colonies of termites collaborate to build termite mounds with integrated ventilation mechanisms to protect the colony from critical temperatures. All these systems accomplish complex tasks through simple local interactions amongst themselves, collectives of autonomous agents, and are commonly referred to as examples of swarm intelligence. Swarm robotics can be seen as a research area that emerged from the field of swarm intelligence, whereas swarm intelligence depicts a subfield of artificial intelligence. The first swarm robotic system was the Khepera robot in 1994 (Mondada et al., 1994). Since then, many other swarm robotic systems have been built, most of them are listed in Table 1. However, much of swarm intelligence research has been conducted via computer simulations. Brambilla et al. analysed more than 60 publications that dealt with swarm robotic collective behaviours. They found that more than half of these publications presented results which were obtained through simulations or models (Brambilla et al., 2013). Although simulators are a valuable tool for exploring, systematically, the algorithmic-behaviour of natural swarms, they frequently involve simplifications and reductionist axioms to enable computational tractability. Such simulated systems can fail to faithfully reproduce the intricate physical interactions and variability that exist in real systems, and their fidelity to the real world is difficult to verify or improve without feedback from physical experiments (Rubenstein et al., 2014).

**Table 1:** Comparison of 13 swarm robotic systems’ sensing capabilities.

**Robot System / Number of Measurands**	2	**3**	**4**
Khepera (Mondada et al., 1994)	IR		
Alice (Caprari and Siegwart, 2003)	IR		
SBot (Mondada et al., 2003)	L		
Jasmine (Kornienko et al., 2005b)	IR		
**E-puck** (Mondada et al., 2009)	IR, L		**AC**
MarXbot (Bonani et al., 2010)	IR, L		
Kilobot (Rubenstein et al., 2012)	IR		
R-One (McLurkin et al., 2013)	IR		
**Droplet** (Farrow et al., 2014)		**IR**	
GRITSBot (Pickem et al., 2015)	IR		
Pheeno (Wilson et al., 2016)	AC		
**HoverBot** (Nemitz et al., 2017)		**MF**	

IR: Infrared Light, AC: Acceleration, L: Visible Light, F: Force, EMF: Electromagnetic Field, MF: Magnetic Field. Other systems that were considered but ended up using “single-measurand sensors”: Swarm Bot (McLurkin et al., 2006), Kobot (Turgut et al., 2007), and Thymio-II (Riedo et al., 2013).

Over the past two decades, many swarm robotic systems have been developed. They all differ in certain aspects such as power consumption, locomotion strategy, or sensing capability. The sensing capabilities of a robot influence the type of experiments one can perform. While a camera adds more functionality to a robot than an ambient light sensor, each sensor comes at a different cost. We systematically analysed previous swarm robotic systems and found that some systems possess sensors that have been, or could be, used for the detection of multiple signals. For example, an IR transceiver could be used for communication and proximity sensing amongst others. Developing robots that are low-cost and functional is a challenging task, therefore, utilising sensors for the detection of multiple measurands is desirable. We believe that this concept is very important and deserves further evaluation.

In the following section, we introduce an *instrument* model, a well-established model borrowed from the sensors community to generally describe a measuring device, to establish a clear understanding of sensors and how they can become multi-functional. Then we will give a comprehensive review on the sensing capabilities of previous swarm robotic systems and categorize them based on their multi-functionality. Finally, we show an implementation in which a HoverBot (Nemitz et al., 2017) extracts multi-functionality from a single Hall-effect sensor. We show that we can associate time-based magnetic field measurements to robot rotation, collision, and successful movement; and we show how to build a corresponding time-based classifier that can be trained offline before it is transferred to HoverBot for online classification. We apply our method to the HoverBot platform and discuss its applications to time-series data.

### 1.2. Instrument Model

**Figure 1:**
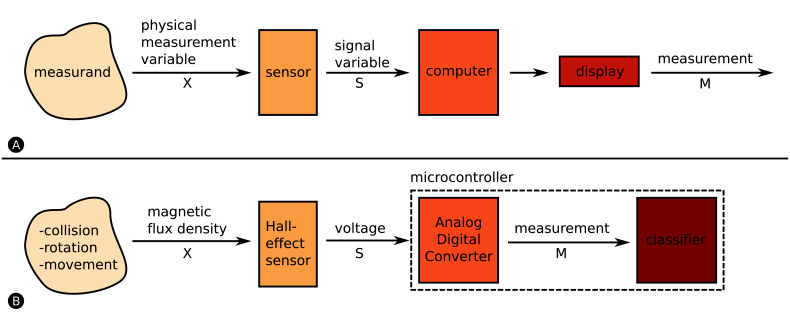
Instrument Model (Webster, 1999). **(A)** The measurand is the to-be-measured value of interest, whereas the physical measurement variable is associated, either directly or indirectly, to the measurand. The sensor converts the physical measurement variable in a signal variable (often an electric signal), and feeds it into a processing unit or computer. The value that we actually display is the measurement. **(B)** Analog to A. We detect collision, rotation and movement measurands by measuring magnetic flux density. The Hall-effect sensor converts the magnetic field into a voltage which is converted through the microcontroller’s ADC to a digital signal. We finally apply the measurements to a classifier which associates the measurements to one of the three measurands.

The *instrument model* shown in Figure 1 is a scientifically accepted model from the sensor community (Webster, 1999) to generally describe a measuring device. An instrument is a device that transforms a physical variable of interest, the *measurand*, into a form that is suitable for recording, the *measurement*, as conceptually shown in Figure 1A. An example of a basic instrument is a ruler. In this case the measurand is the length of some object and the measurement is the number of units (meters, inches, etc.) that represent the length.

Any measurand (distance, collision, temperature, etc.) is linked to an *observable physical measurement variable* X. The observable physical measurement variable X does not necessarily have to be the measurand, X can only be related to the measurand. For instance, the mass of an object is often measured by the process of weighing, where the measurand is the *mass* but the physical measurement variable is the downward *force* the mass exerts in the Earth’s gravitational field. Collision detection is another example. A robot can detect the measurand *collision* by measuring *force* or by relating the measurand to another physical measurement variable such as *acceleration*. In this case you can either purchase a single accelerometer or a set of force sensors (e.g., four force sensors – one sensor on each robot side). Both implementations allow the detection of collisions, however, the single accelerometer is likely going to be cheaper than the force sensors. There are many more of such examples, but there are also variants in which a single physical measurement variable contains information about several measurands. An excellent example is IR light. IR light can be used for the measurement of distance, to determine bearing and to communicate with other robots as shown by (Farrow et al., 2014). We call this capability *multi-functional sensing*. Communication is usually handled by a transceiver; you transmit and receive or *transceive* data by means of a physical channel e.g., by utilising electromagnetic waves in the IR spectrum. The *receiving* of signals requires sensors, such as photodiodes that transduce IR light into electric signals, hence communication itself can be considered as a sensing task.

### 1.3. Sensing Capabilities of Swarm Robotic Systems

We reviewed the sensing capabilities of 15 swarm robotic systems found in the literature and we summarize our findings in Table S1 of the supplemental material. Table 1 is a subset of Table S1 containing robot systems capable of multi-functional sensing with ≥2 measurands. The content of Table 1 is based on the cited work shown in the first column of each row. Table 2 takes an even closer look at the few robot systems capable of multi-functional sensing with ≥3 measurands.

**Table 2:** Further comparison of swarm robotic systems with 3 or more measurands per sensor.

**Measurand / Physical Measurement Variable**	IR	AC	MF
	**Droplet**	**E-puck**	**HoverBot**
Local Communication	X		
Proximity/Distance	X		
Bearing	X		
Inclinometer		X	
Collision		X	X
Free-fall		X	
Movement acceleration		X	
Odometry			X
Rotation			X

IR: Infrared Light, AC: Acceleration, MF: Magnetic Field

The majority of swarm robotic systems (12/15) are capable of multi-functional sensing with ≥2 measurands. In the ≥2 measurand category, the most commonly used *physical measurement variables* are IR light, followed by ambient light. IR light has been mainly utilised for distance/proximity sensing and local communication, whereas ambient light has been often utilised for object detection and long-range distance measurements through a camera.

To the best of our knowledge, there are only two swarm robotic systems that are capable of, or make use of, multi-functional sensing with ≥3 measurands. The e-puck is capable of measuring four measurands with a single sensor (Mondada et al., 2009). It uses a single accelerometer to measure *inclination*, *collision*, *free-fall* and *movement acceleration*. The Droplet is capable of measuring three measurands with a set of IR sensors (Farrow et al., 2014). It uses six symmetrically placed IR sensors to measure *distance*, *bearing* and local *communication.* Therefore, once a robot possesses an accelerometer and a group of IR sensors, it is capable of measuring seven measurands by only using two different types of sensors.

**Figure 2:**
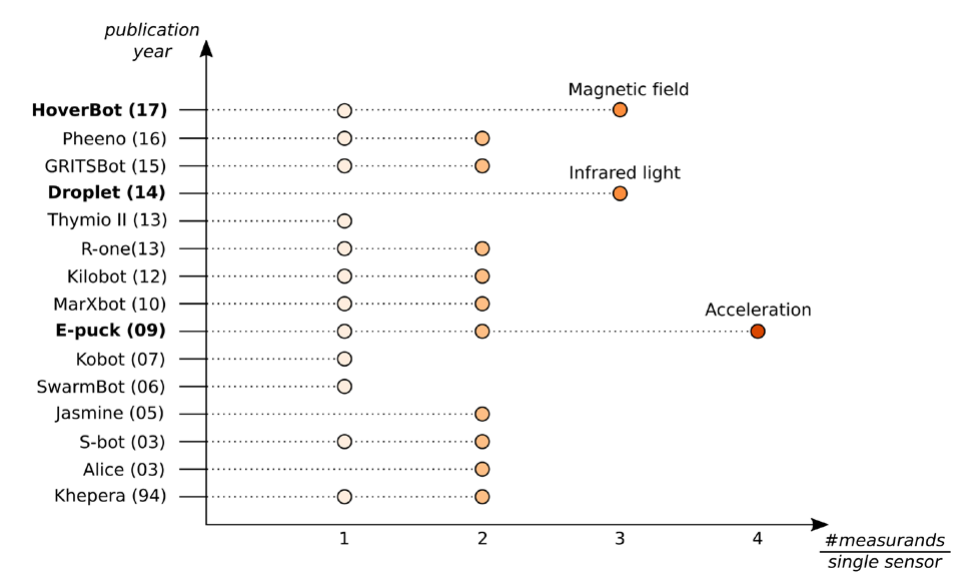
Overview of the sensing capabilities of previous swarm robotic systems. Swarm robots are sequentially listed according to their publication date. The x-axis indicates how many measurands can be measured per single sensor. Although most swarm robotic systems utilise their sensors to detect up to two measurands, there are only three robot systems including the HoverBot that utilise their sensors to measure 3 or more measurands.

Our work, which we present here, adds another system to the ≥3 measurand category. Figure 1B indicates HoverBot’s instrument model. The measurands *successful movement, collision* and* rotation* can be related to the physical measurement variable *magnetic flux density*. HoverBot uses a Hall-effect sensor to convert the magnetic flux densities into voltages which are subsequently converted via an analogue digital converter to digital measurements. The microcontroller processes the samples and checks them against a previously trained classifier. Our classifier combines dynamic time warping and barycenter averaging to build time-variant representations of the measurands. Figure 2 gives an overview of all swarm robotic systems and their sensing capabilities.

The instrument model comes with its limitations: the *measurand* component can be interpreted from different angles. For example, successful movement and rotation can be generally considered as being part of a robot’s *odometry* capability. However, the to-be-measured value, the measurand, is not odometry but successful movement and rotation. Since the instrument model is to some extent subjective, it is of paramount importance to apply this model consistently. Figure 2 is a collection of carefully categorised sensing capabilities of swarm robotic systems. A key understanding that we have derived from studying the instrument model is that, in swarm robotic systems, often the sensors could be further utilised, and therefore the systems should be reanalysed.

### 1.4. Applying Multifunctional Sensing

Our work empirically shows how HoverBot’s single Hall-effect sensor can be augmented for the detection of *collision*, *rotation*, and *successful movement*. Although we specify in the discussion under which circumstances our techniques might be applied to other sensors, we would like to give a brief insight into the research opportunities that potentially arise from (i) HoverBot’s new sensing capabilities and (ii) our approach more generally.

#### 1.4.1. Collision Dependent Behaviours

*Rotation* and *successful movement* detection are proprioceptive sensing capabilities, hence give insight into the internal state of a robot. Counting successful movements is useful for the robot to keep track of its position (odometry), and detecting rotations to derive knowledge about its orientation. Detecting *collisions* is an exteroceptive sensing skill. It provides the robot with information about its surroundings. We give a brief overview over the few swarm robotic studies that deal with collisions and indicate how they were utilised. Kernbach et al. and Schmickl et al. worked on the re-embodiment of biological aggregation behaviours of honeybees. They show how to take advantage of collisions to develop scalable robot behaviours. In their work, swarm robots converge to light sources without requiring inter-robot communication. Concretely, they minimize sensing and computation by evaluating robot data only once per *collision*; more frequent *collisions* lead to more data evaluations (Kernbach et al., 2009; Schmickl et al., 2009). Mayaa et al. harnessed collisions to help localise a robot within an arena. The arena was divided into differently sized segments, whereas each segment was inhabited by differently sized robot groups. Robots used collision detection as information source to determine their locations (Mayya et al., 2017).

Overall, *collision* is a promising candidate for research on and the design of *scalable* robot behaviours since collisions incidences usually increase with increasing group sizes. *Scalable* refers to the ability of a swarm to perform well with different group sizes; the introduction or removal of individuals does not result in drastic change in the performance of a swarm (Brambilla et al., 2013). Collisions have only been sparely studied in the swarm robotics context. Kernbach, Schmickl and Mayaa et al.’s work depict excellent starting points for future work on collisions; the HoverBot system depicts a suitable research platform since it embraces collisions and is capable of detecting them.

#### 1.4.2. Collective Perception

Other interesting work that might profit from our approach is research on collective perception. Collective perception broadly refers to collectives that explore an environment and evaluate its features (Valentini et al., 2016). The work presented here has the potential to enhance a robot’s sensing capabilities without modifying its hardware, hence, could add to the list of observable features for collective perception. Notable literature on collective perception includes Khaluf’s work on detecting and marking features e.g., of pollution areas (Khaluf, 2017), Kornienko et al.’s work on investigating which sensing and processing steps should be done individually or collectively for collective perception with robot swarms (Kornienko et al., 2005a), Schmickl et al.’s work on hop-count and Trophallaxis-inspired strategies to collectively perceive targets (Schmickl et al., 2007), Mermoud et al.’s work on aggregation-based strategies to collectively perceive and destroy specific targets (Mermoud et al., 2010), and Tarapore et al.’s work on collective perception strategies inspired by the adaptive immune response to discriminate between dangerous and friendly cells (Tarapore et al., 2013).

## 2. The HoverBot System

### 2.1. Review

**Figure 3:**
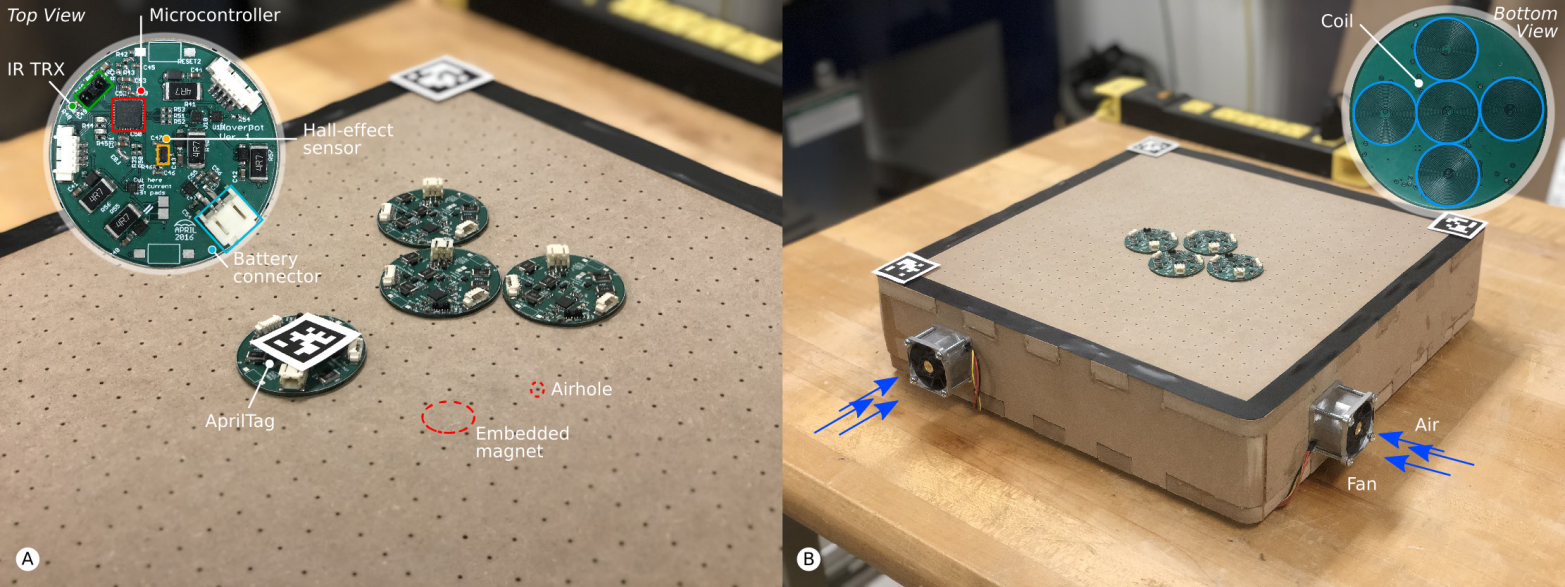
The HoverBot system. **(A)** The HoverBot is displayed in detail in the top left corner. It consists of a low-cost microcontroller, an IR transceiver and a Hall-effect sensor. Permanent magnets are embedded into the platform and air holes are drilled through the surface as exemplary indicated through red circles. We placed AprilTags on a HoverBot as well as in three of the four corners of the magnet-levitation table. This setup allows us to keep track of HoverBot’s position during experiments. **(B)** The bottom side of the HoverBot is displayed in the top right corner. A HoverBot possesses five planar coils that it uses to manoeuvre two-dimensionally on the magnet-levitation table. We installed four fans, one on each side of the levitation-magnet table. The fans force air into the magnet-levitation table creating a pressure differential between the inside and outside of the table. Air streams through the porous surface of the magnet-levitation table creating air-cushions beneath HoverBots which makes the robots levitate.

We reported in (Nemitz et al., 2017) on the HoverBot system. The HoverBot system is a swarm robotic system and the first of its kind that uses *active low-friction locomotion*. Active low-friction locomotion supplies robots with a constant air flow beneath their surfaces. The airflow causes a reduction of friction between robot and arena surface allowing relatively weak forces to be used for locomotion. In addition, we embedded permanent magnets into the arena as indicated in Figure 3A. The HoverBot is a levitating circuit board that possesses planar coils that interact with the arena magnets, resulting in two-dimensional locomotion. Such forces would be insufficient if friction had not been reduced. From the outset, the HoverBot system was designed for manufacturability: HoverBots only require electronics components that are surface mountable, only require connecting a battery to a robot as an assembly step, use low-cost actuators and associated circuitry, do not require actuator calibration and move precisely on a discrete grid. For more details, please refer to our publication (Nemitz et al., 2017).

The work presented here focuses on HoverBot’s sensing capabilities. The HoverBot is equipped with IR and Hall-effect sensors as shown in Figure 3A. The IR sensor points upwards and therefore only allows communication to an overhead IR handheld that is connected to a PC rather than to other robots. The Hall-effect sensor is positioned in the centre of the HoverBot agent and measures the ambient magnetic flux density. Simplified, a Hall-effect sensor is a transducer that converts a magnetic field into a voltage difference, whereas magnetic fields, also called magnetic flux densities, are measured in Tesla [N/m^2^] or Gauss [1T = 10,000G].

### 2.2. The Magnetic Field

**Figure 4:**
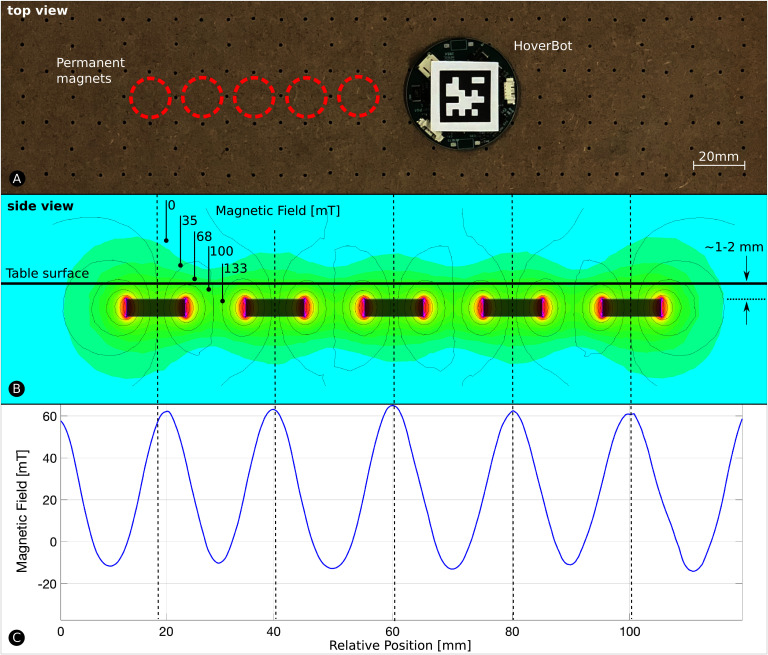
The Magnetic Field. **(A)** HoverBot moves on its magnet-levitation table. **(B)** We simulated HoverBot’s magnetic field measurements via finite element method. The permanent magnets are embedded into the magnet-levitation table and are manufactured to be ~1–2 mm beneath the surface. **(C)** We took magnetic field measurements from a HoverBot during movement.

HoverBots energize their coils to pull themselves towards magnets. We simulated and subsequently took measurements from the magnetic field. Figure 4B depicts the simulated magnetic field acquired with FEMM, a simulator for solving low frequency electromagnetic problems on two-dimensional planar and axisymmetric domains (Meeker, 2010). The grey rectangles in Figure 4B indicate magnets and the dashed lines serve as reference to the measurements in Figure 4C.

We obtained the magnetic field in Figure 4C with a HoverBot. The simulated and measured magnetic fields broadly correlate with one another. The first amplitude in Figure 4C is slightly shifted due to fabrication tolerances. The pocket holes of the permanent magnets are slightly larger in diameter than the magnets themselves leading to imperfect magnet alignments. For more details on the manufacture of the HoverBot system please see our previous paper (Nemitz et al., 2017).

### 2.3. Magnetic Field Profiles

**Figure 5:**
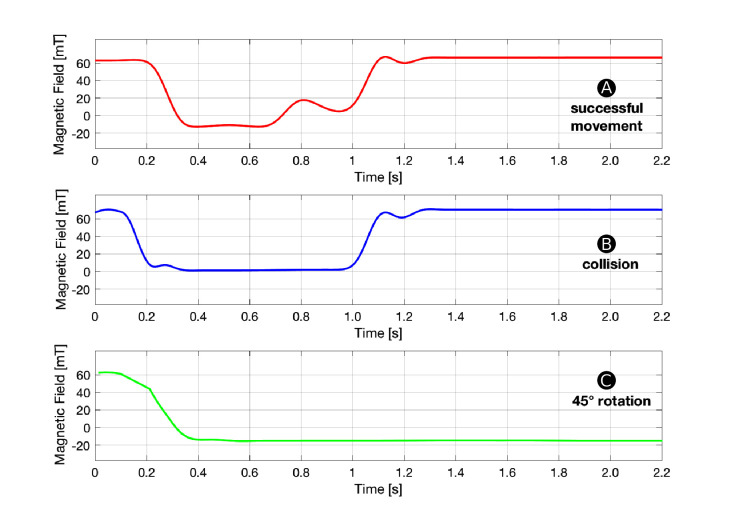
Magnetic Field Profiles. This figure shows examples of magnetic field measurements over time that a HoverBot measures during **(A)** a successful movement **(B)** a collision and **(C)** a 45-degree rotation. Each time series is distinctly different from the other.

During operation, a HoverBot agent measures magnetic field values in the range of approximately −20 to +60 mT as shown Figure 4C. However, the actual measurements during movement look somewhat different since they are not only *position-dependent* as in Figure 4C but also *time-dependent*. Figure 5 shows a set of time-dependent magnetic field measurements. Figure 5A indicates the magnetic field measurements of a HoverBot during a successful movement from one permanent magnet to another. When a HoverBot agent collides with an object, its magnetic field measurements look distinctly different compared to its successful movement measurements as shown in Figure 5B. We reported in (Nemitz et al., 2017) that HoverBot could potentially lose their orientation, rotate 45 degrees, and lock into position. Figure 5C indicates the magnetic field measurements that occur in this incident.

The magnetic field profiles for successful movement and collision in Figure 5A,B possess distinct magnetic field changes over time. The magnetic field profile for rotation in Figure 5C differs from the other profiles since the magnetic field does not change anymore once the robot rotated and locked into position. Therefore, detecting rotation is a simple case of measuring a constant negative magnetic field over time. Since detecting rotation is trivial and does not add value to this work, we explicitly decided to focus on the remaining more challenging profiles. The remaining work focuses on the classification of time series data for successful movements and collisions.

### 2.4. Data Acquisition

One of HoverBot’s advantages is its precise locomotion in Manhattan Geometry (Nemitz et al., 2017). Missteps or rotations are rare events. During data acquisition, we only observed successful movements and collisions. The robot randomly moved within the arena and occasionally collided with arena boarders. Our dataset consists of 259 samples, whereas 203 samples are from successful movements and 56 samples are from collisions. Each sample is a collection of timestamps, magnetic field measurements, x-position, y-position and orientation of the HoverBot. We acquired our data with the experimental setup in (Nemitz et al., 2017). We placed an artificial fiducial, an AprilTag (Olson, 2011), onto the HoverBot. AprilTags are simplified 2D barcodes that are robust to occlusions and lens distortion allowing high detection rates with camera systems - 20 Hz in our setup. To measure the trajectory of a HoverBot, we tracked the centroid and the orientation of the robot’s AprilTag. We used a Chameleon 1.3 MP Color (Sony IXC445) camera and a Tamron 13FM28IR 2.8 mm f/1.2 day/night lens. We also installed an IR transceiver above the arena and connected it to a centralised PC to record HoverBot’s magnetic field measurements; HoverBot transmitted during runtime its magnetic field measurements *online* to the overhead IR transceiver. The camera system and the IR transceiver were embedded into LCM (Huang et al., 2010) providing us with a robust data acquisition platform for our experiments.

## 3. Time Sequence Classification

A major challenge in discriminating between successful movement and collision profiles is their variations in the time and measurement domain. For example, a HoverBot’s speed may vary between actuations, resulting in measurement signals that are stretched or compressed. Additionally, environmental factors can cause measurement values to vary over time, such as when HoverBot’s elevation above the table (and hence, its position in the magnetic field) varies due to air pressure fluctuations in the magnet-levitation table. To classify the measurement profiles depicted in Figure 5A,B, we must account for these variations while operating within the computational constraints of HoverBot’s microcontroller.

**Figure 6:**
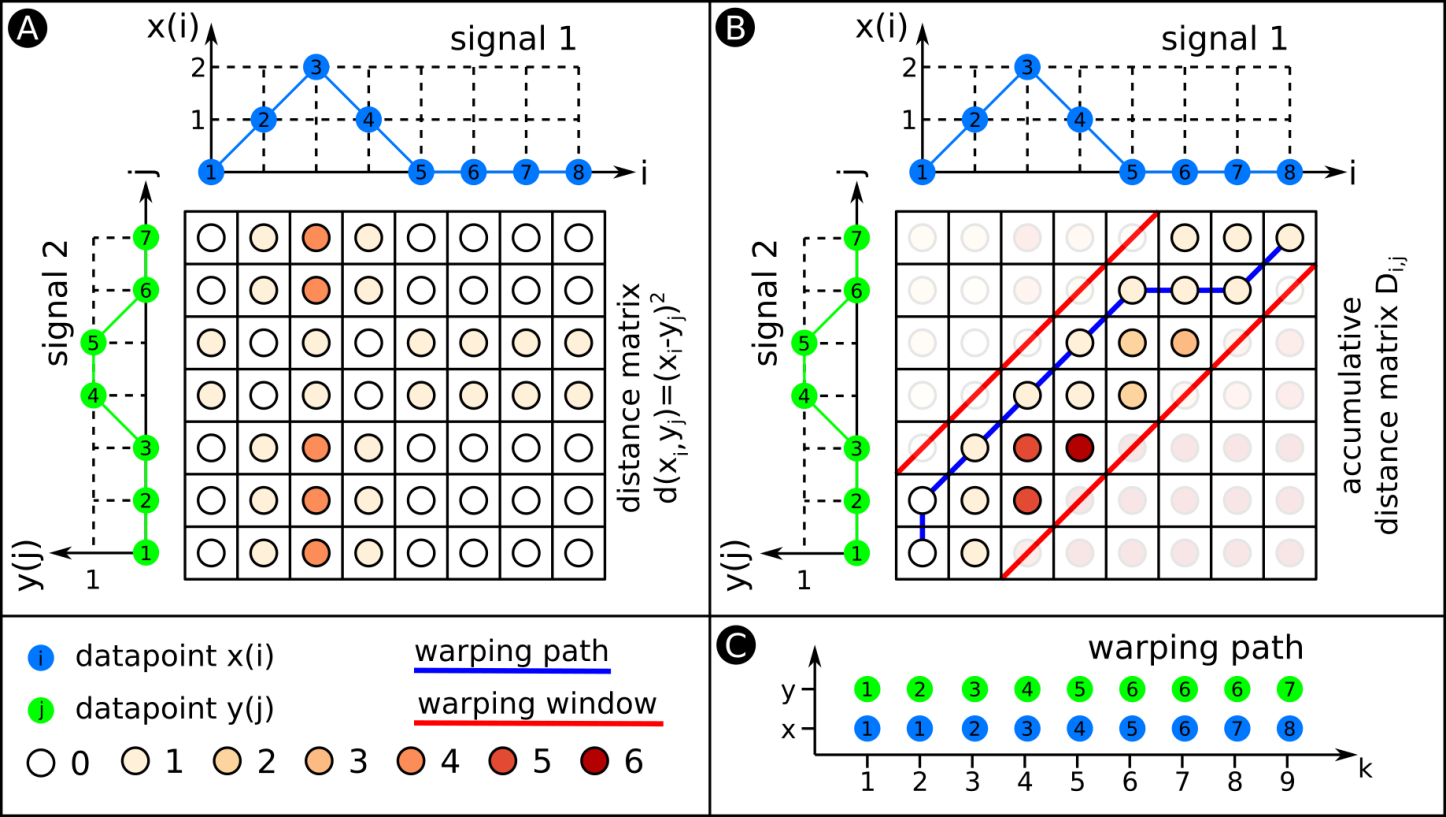
Dynamic Time Warping (DTW). This figure exemplarily explains DTW. **(A)** We compute a matrix in which each entry is the Euclidean distance between datapoints from signals 1 and 2. The colour code for the various distances is shown below. **(B)** We use the distance matrix from **(A)** to develop the accumulated distance matrix D. The two red lines indicate the warping window. The warping path illustrated as blue line has to fit within the borders of the warping window. **(C)** The warping path explicitly states which datapoints of signal 1 align with what datapoints of signal 2. The warping path is always at least as long as the longest signal that is warped. In this example, the warping path exceeds both signals’ length, the warping path is 9 datapoints long.

**Figure 7:**
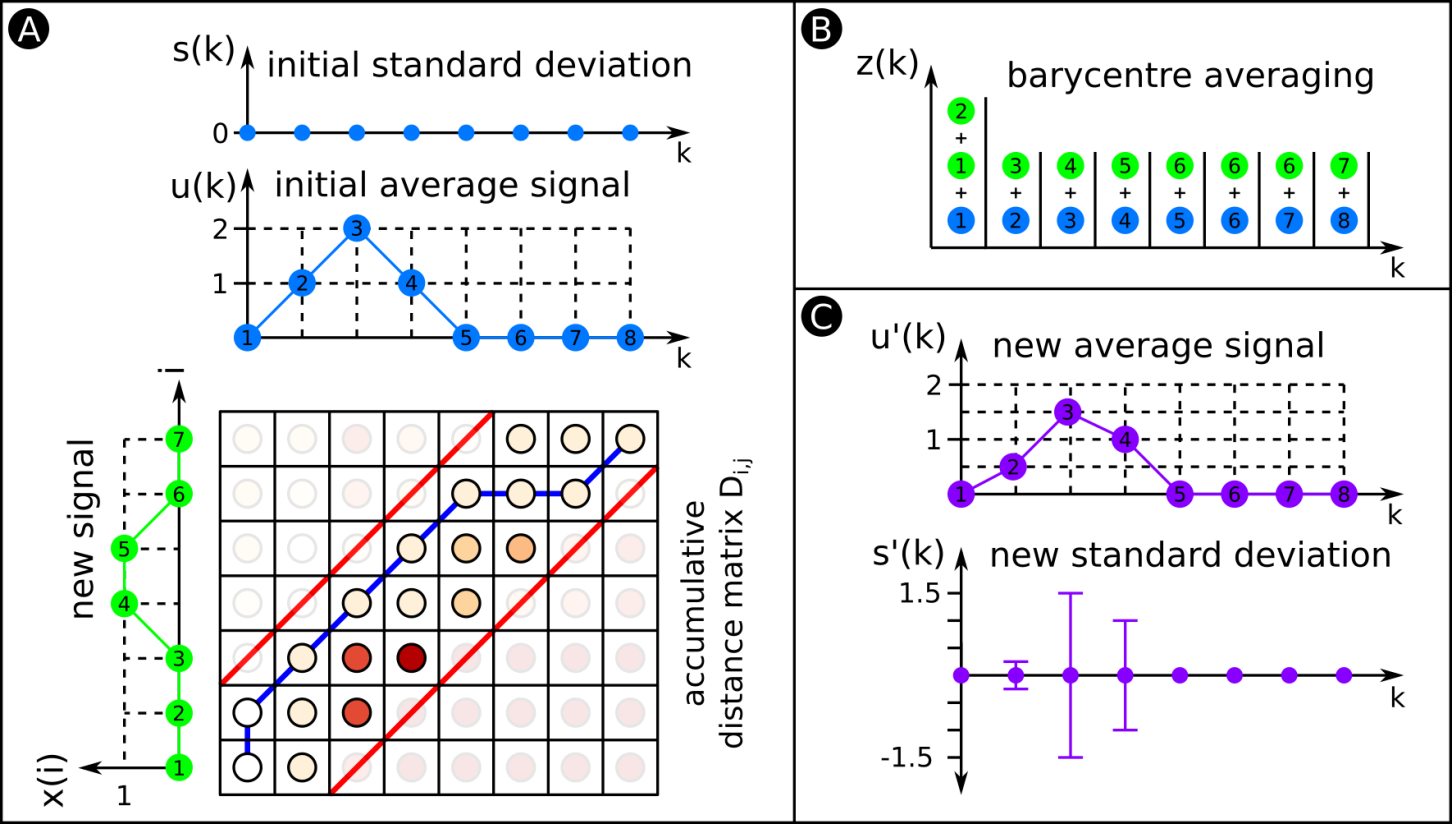
DTW Barycentre Averaging (DBA). **(A)** Similar to Figure 6B. This time the signals are initial average signal u(k) and new signal x(i), whereas x(i) will be aligned and averaged with u(k). **(B)** DBA performs DTW to align both signals. The warping path indicates which datapoints from x(i) are aligned with what datapoints from u(k). There is a container for each k; we copy the datapoints that are warped to a specific k (see Figure 7A) into the corresponding container. **(C)** We compute from the datapoints of each container the average signal and SD. To build the average signal and SD of a group of signals, warp all signals with the initial average signal. This process fills the various containers with many more datapoints, however, the technique remains the same.

We describe in the following sections a classification method that learns offline representations of each measurement profile and stores these representations on the robot. When the HoverBot agent obtains a new series of measurements online, the measurements are compared to the stored class representations to determine the maximum likelihood classification. Our classification procedure builds on several component techniques from the field of signal processing. We introduce each of these components in turn in Section 3.1 and then incorporate them into our method in Sections 3.2 and 3.3. Throughout Section 3.1 we refer to Figures 6 and 7 for specific examples.

### 3.1. Classification Preliminaries

#### 3.1.1. Dynamic Time Warping (DTW)

Dynamic Time Warping (DTW) (Chiba and Sakoe, 1978) is used to align two time sequences of potentially different length and measure the amount of similarity between them. DTW finds correspondences between points from the two sequences by warping them in the time domain. Given a distance measure, DTW computes the set of point correspondences that minimizes the cumulative distance between the sequences. Figure 6 shows an example of DTW for two arbitrary signals.

Consider two input signals $x=\left[x_{1},\cdots ,x_{M}\right]$ and $y=\left[y_{1},\cdots ,y_{N}\right]$. Let $d(x_{i},y_{j})$ be a measure of the distance between $x_{i}$ and $y_{j}$, such as the squared Euclidean distance $d\left(x_{i},y_{j}\right)={\left(x_{i}-y_{j}\right)}^{2}$. First, DTW computes the *distance matrix* d (Figure 6A) and subsequently the *accumulated distance matrix* D (Figure 6B). DTW initialises the first row and column of D as follows:

$$D_{1,1}=d\left(x_{1},y_{1}\right)$$

$$D_{i,1}=D_{i-1,1}+\;d\left(x_{i},y_{1}\right),\;i=2,\cdots ,M$$

$$D_{1,j}=D_{1,j-1}+\;d\left(x_{1},y_{j}\right),\;j=2,\cdots ,N$$

The rest of D is then computed as:

$$D_{i,j}=d\left(x_{i},y_{j}\right)+\min\left\{ \begin{array}{l} D_{i-1,j}\\ D_{i,j-1}\\ D_{i-1,j-1} \end{array}\right.\;,\;\;\begin{array}{c} \;i=2,\cdots ,M\\ j=2,\cdots ,N \end{array}$$

The DTW algorithm uses dynamic programming to build D, where each element

$D_{i,j}$ gives the minimum cumulative distance between subsequences $\left[x_{1},\cdots ,x_{i}\right]$ and $\left[y_{1},\cdots ,y_{j}\right]$. The final element of D, $D_{m,n}$, represents the minimum cumulative distance between the signals under any warping configuration, thus serving as a measure of similarity between the signals. Such an optimal warping configuration is given by the warping path $\phi^{x,y}=\left[\left(\phi_{1}^{x},\phi_{1}^{y}\right),\cdots ,\left(\phi_{K}^{x},\phi_{K}^{y}\right)\right]$ through matrix D (Figure 6C). Each element $\phi_{k}^{x,y}=\left(\phi_{k}^{x},\phi_{k}^{y}\right)$ contains an index $\phi_{k}^{x}$ of signal x that has been matched to index $\phi_{k}^{y}$ of signal y. The warping path can be computed as:

$$\phi_{K}^{x,y}=\left(M,N\right)$$

$$\phi_{k}^{x,y}=\underset{i,j}{argmin}\;D_{i,j}\;i,j=\left\{ \begin{array}{l} \phi_{k+1}^{x}-1,\phi_{k+1}^{y}\\ \phi_{k+1}^{x},\phi_{k+1}^{y}-1\\ \phi_{k+1}^{x}-1,\phi_{k+1}^{y}-1 \end{array}\right.,\;k=K-1,\cdots ,2$$

$$\phi_{1}^{x,y}=\left(1,1\right)$$

The length K of warping path $\phi^{x,y}$ varies depending on the extent to which the signals are warped, but it will satisfy the inequality max(M,N)≤K≤M+N−1.

#### 3.1.2. Constrained Dynamic Time Warping (CDTW)

The DTW formulation of the previous section is *unconstrained* i.e. the algorithm considers any possible warping configuration in matrix D. This can lead to *pathological* warping configurations wherein a single point from one signal is matched with many points from the other signal. Unconstrained DTW is thus sensitive to spurious alignments between signals. For example, unconstrained DTW might warp the collision time series in Figure 5B onto the successful movement time series in Figure 5A even though the signals correspond to disparate events. Given these limitations of unconstrained DTW, it is common to limit the extent to which signals can be warped in the time domain. In Constrained Dynamic Time Warping (CDTW), we allow correspondences between points only if those points occur within a fixed time period of one another. The length of the warping window W is application-dependent and defined as |n−m|≤W. W determines how many elements of matrices d (distance matrix) and D (accumulated distance matrix) are calculated. We show an example of a warping window in Figure 6b. CDTW can offer benefits in terms of time- and space-complexity, both are important for embedded platforms like the HoverBot. Because the warping path is constrained, only a portion of the matrix D must be computed and stored (Chiba and Sakoe, 1978). As a result, the time- and space-complexity are both reduced from O(N∙M) to O(N∙W), where the length of the warping window W is much less than M, the length of signal y.

#### 3.1.3. DTW Barycenter Averaging (DBA)

For a classification task, it is often useful to compute a summary representation of a class of data. This averaging process is non-trivial when performed on variable-length signals. Naïve approaches based on pairwise alignment and averaging are sensitive to ordering effects and produce prohibitively long alignment sequences (Petitjean et al., 2011). We instead utilize DTW Barycenter Averaging (DBA) to compute the average signal μ and the standard deviation σ of a group of signals (Morel et al., 2018). Figure 7 shows an example of DBA for *two* arbitrary signals.

Consider a group of signals X to be input to the DBA algorithm. DBA initializes σ to be zero and randomly selects a signal $x^{\left(0\right)}=\left[x_{1}^{\left(0\right)},\cdots ,x_{K}^{\left(0\right)}\right]$ to serve as the initial average signal μ (Figure 7A) as

$$\mu_{k}=x_{k}^{\left(0\right)},\;k=1,\cdots ,K$$

DBA also initializes sets $\psi_{k}=\emptyset ,k=1,\cdots ,K$ that are used in the average computation. Each set $\psi_{k}$ contains $z\in \psi_{k}$ elements for each specific k∈K, whereas z is a placeholder for aligned datapoints (Figure 7B). Each signal $x\in X\setminus x^{\left(0\right)}$ is aligned with the average signal using CDTW. Each set $\psi_{k}$ is updated as

$$\psi_{k}=\psi_{k}\cup \left\{x_{\phi_{i}^{x}}\;:\;\phi_{i}^{\mu }=k\right\}$$

Please note that our example in Figure 7 only shows DBA for *two* signals. In Figure 7B, set $\psi_{1}$ consists of three aligned datapoints and sets $\psi_{2-8}$ of two aligned datapoints. Once this process has been repeated for *all* signals, the value of each element of the average signal is updated as the barycenter of all points that map to the corresponding element of the existing average signal μ (Figure 7C); that is,

$$\mu_{k}=\frac{\sum_{z\in \psi_{k}}z}{\left|\psi_{k}\right|}$$

Likewise, the elements of the standard deviation σ (Figure 7C) are updated as

$$\sigma_{k}=\sqrt{\frac{\sum_{z\in \psi_{k}}{\left(z-\mu_{k}\right)}^{2}}{\left|\psi_{k}\right|}}$$

The resulting updated average signal retains its original length while incorporating information from the entire group of signals. The process of aligning the group of signals with the average signal and computing an updated average and SD can be repeated multiple times for better convergence.

#### 3.1.4. Downsampling

Our method can be implemented in several variants. Unconstrained with a complexity of O(N∙M), constrained with a complexity of  O(N∙W) using a warping window, or even further constrained with a complexity of O(K∙W) through a combination of warping window and downsampling. Consider an input signal $x=\left[x_{1},\ldots ,x_{N}\right]$ with length N. We reduce the signal’s length from N to K by dividing the signal x into K parts, each of which contains $L=\frac{N}{K}$ measurements. The value of each element $x_{k}^{\prime }$ of the downsampled signal $x^{\prime }$ is given by

$$x_{k}^{`}=\frac{1}{L}\sum_{i=L\left(k-1\right)}^{Lk}x_{i},k=1,\ldots ,K$$

Downsampling has an impact on the classifier’s detection rate. The intuition is, the more you downsample the input signals, the worse the detection rate becomes. Downsampling potentially averages out important signal features that otherwise helped the classifier to discriminate between signals. From an engineering perspective, the more you downsample the input signals, the less memory is required to store values of the cumulative distance matrix. Memory reductions are very useful since it lowers the requirements for our low-cost microcontroller. Hence, there is a tradeoff between classification performance and memory utilization. We are able to achieve high classification performance with acceptable memory usage as shown in the Result section.

### 3.2. Offline Learning of Class Representations

**Figure 8:**
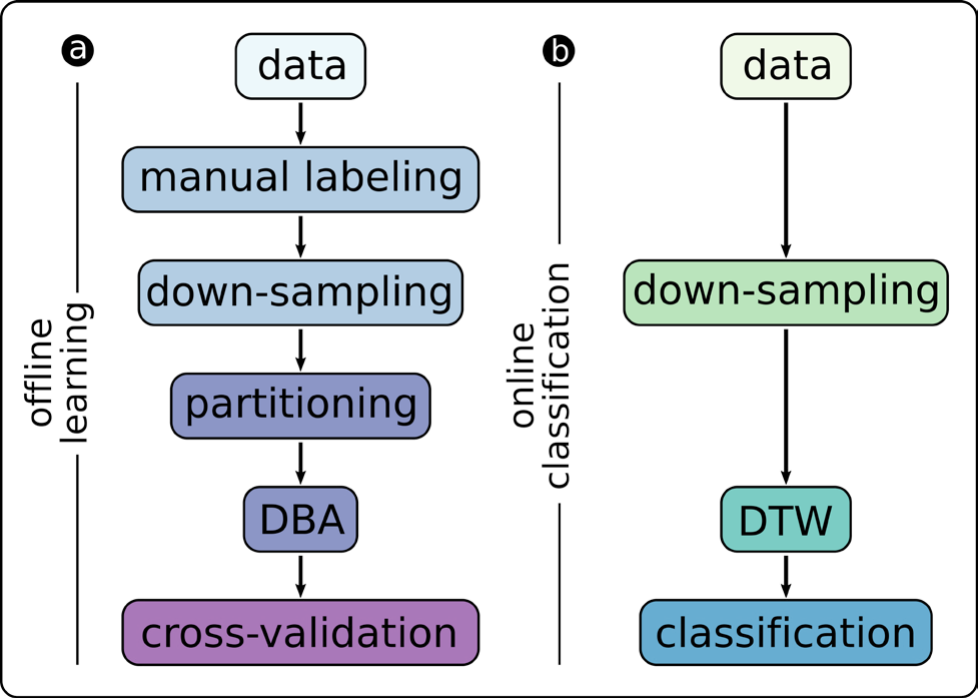
Overview of signal processing components for **(A)** offline training of the classifier and **(B)** online classification of new data samples.

Figure 8A gives an overview of the components that are involved in the offline learning of class representations. First, we conduct a random-walk HoverBot experiment and record the measurements (x-, y-position, orientation, magnetic field measurement, timestamp). We separate the data into approximately two second intervals, HoverBot’s coil actuation scheme, to obtain a dataset consisting of many training examples. We manually label these examples based on HoverBot’s movement over time. We also downsample (K=20) each example to accord with the computational limitations of HoverBot’s microcontroller.

Given this set of labelled training examples, we perform k-fold cross-validation (KV=10). In each iteration, we divide (partition) the training data into labelled classes and perform DBA to obtain a representation of each class. In the validation step, we compute the Mahalanobis distance between each held-out example and the representations of each class:

$$d\left(\mu_{k},\sigma_{k},x_{k}\right)=\sqrt{\sum_{k=1}^{K}\frac{{\left(x_{k}-\mu_{k}\right)}^{2}}{\sigma_{k}}}$$

The Mahalanobis distance measures the distance between a point and a distribution (De Maesschalck et al., 2000). In other words, how many standard deviations σ is a point x away from the mean value μ. We classify each example according to the minimum-distance class, which corresponds to the maximum-likelihood classification.

### 3.3. Online Classification of Hall-Effect Measurements

Figure 8B indicates the components that are involved in the online classification. The components in Figure 8B are a subset of Figure 8A. HoverBot tries to move into a direction and records magnetic field measurements. Depending on the downsampling frequency, the HoverBot stores a number of average values into its memory (K = 20). The new dataset is used to calculate the Mahalanobis distance for each class representation (successful movement and collision). The Mahalanobis distances are compared with one another. If the distances do not reach a minimum value, the event will be labelled as *unknown*. Otherwise the event will be classified according to the lower Mahalanobis distance. The parameters of the class representations ($\mu_{k},\sigma_{k}$) were trained offline, do not change during runtime and therefore are stored on the microcontroller’s read-only-memory (ROM). The amount of ROM memory involved is dependent on the downsampling frequency.

## 4. Results

**Figure 9:**
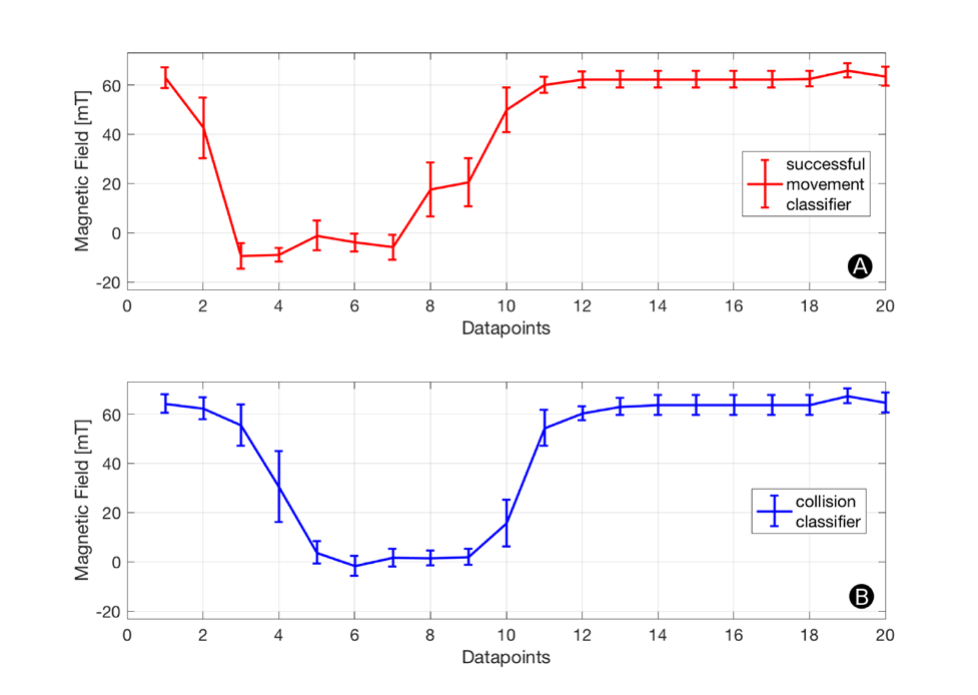
Classifier Parameters. Each datapoint has a mean value and SD associated to it. These constants are stored in the microcontroller’s ROM and used for online classification. The graphs indicate how much tolerance (SD) is permitted at each datapoint.

Figure 9 shows the trained class representations for successful movement and collision. For each datapoint our classification method produces a mean value and a SD. These values are constant and are stored in the microcontroller’s flash memory.

**Figure 10:**
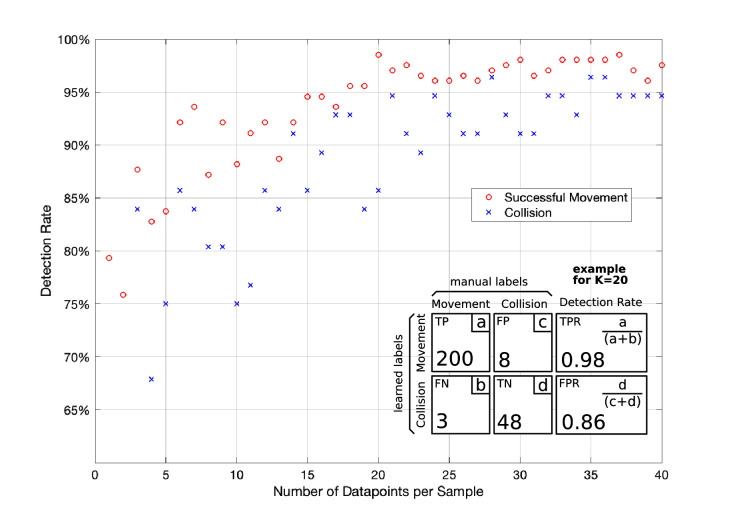
Downsampling. This graph shows the effect of downsampling on the classifier’s detection rate. The detection rate exponentially increases until it starts stagnating once it exceeds 20 datapoints per sample. In the bottom right corner of this figure, we show an example of the confusion matrix for 20 datapoints. Confusion matrix legend: TP: True Positive, FN: False Negative, FP: False Positive, TN: True Negative, TPR: True Positive Ratio, FPR:False Positive Ratio.

Figure 10 indicates the detection rates of our successful movement and collision classifier. For each number of datapoints (K) we compute a confusion matrix through k-fold cross-validation as described in the *Offline Learning of Class Representations* Section. The detection rate for a successful movement is calculated by the True-Positive-Rate and the detection rate for a collision by the False-Positive-Rate of the confusion matrix. We give a confusion matrix example for K = 20 in Figure 10. While the detection rate increases with the number of datapoints, it starts stagnating once it exceeds 20 datapoints per sample. Therefore, we chose K = 20 in our setup achieving collision and successful movement detection rates of greater 85%. This setup is a fair trade-off between detection rate and computational effort. Our microcontroller has to store 20·20·2 Bytes, assuming a 16-bit unsigned integer for each magnetic field measurement and not applying a warping window which otherwise decreased the memory requirement to 20·W·2 Bytes. HoverBot’s microcontroller (Atmel SAMD21E16) comes with 8 kB SRAM which comfortably meets the memory requirements of our classification method.

## 5. Discussion

This work combines two rather unrelated research fields, the fields of signal processing and swarm robotics. The signal processing techniques were specifically adapted to operate on low-cost hardware. We introduced a warping window and a downsampling method to reduce the classifier’s time- and space-complexity. We discuss the impact of downsampling on time varying signals in Section 5.1. We hope that our work encourages other swarm roboticists to reanalyse the sensing capabilities of their robots. The requirements and limitations of our approach are discussed in Sections 5.2 and 5.3. There might be an advantage of adding dedicated general purpose integrated circuits (IC) or Field Programmable Gate Arrays (FPGAs) to robot designs to process sensor data on the side to augment a robot’s sensing capabilities. The advantages and disadvantages of specialized sensory is discussed in Section 5.4.

### 5.1. Downsampling

Our downsampling method decreases a signal’s length to a number of averaged datapoints. HoverBot’s magnetic field profiles for successful movement and collision are simple. The profiles do not contain high frequency features, hence downsampling only had a limited impact on our classifier’s detection rate. Classifiers built upon more complicated time-variant data are expected to be more heavily influenced by our downsampling method.

### 5.2. Applicability to Other Systems

This study has demonstrated how time sequence classification can be used to measure several measurands with a single sensor. Our method’s applicability is dependent on *signatures*, unique measurement profiles that can be associated to specific measurands. Although we have not applied time sequence classification to other sensors or robots, we argue that it is generally applicable if the signature (i) contains time varying measurements (ii) is systematically reoccurring and (iii) is distinctly different to other signatures.

Please find an analysis of suitable sensors, a generalised concept about signatures, and hypothetical examples of our approach in the Appendix.

### 5.3. Limitations

HoverBot’s *discrete movement* helped the discovery that signatures can be used to measure several measurands with a single sensor. If HoverBot’s movement was *continuous*, we could still chop the measurements into the measurement profiles shown in Figure 5, since any continuous movement can be regarded as a finite number of discrete movements with an infinitely small time difference in between them. However, our signatures are constrained to HoverBot’s *movement behaviour*. HoverBot moves on a grid in Manhattan geometry which ensures that it measures a reoccurring magnetic field pattern. Other *movement geometries* such as continuous movements in arbitrary directions would impact our signatures and hence the time sequence classification.

### 5.4. Multifunctional Sensing vs Specialised Sensory

Our *signatures* allow the *binary* detection of successful movements, collisions, and rotations. These measurands can also be measured with specialised sensors. For example, you could detect collisions through tactile sensors that output *continuous* measurements. However, this is not a limitation of the time sequence classification but of our signatures. If you can find *distinct* signatures for each collision level, the time sequence classification will enable you to discriminate between them. The advantage of specialised sensory however is that it takes away the processing behind the time sequence classification and enables continuous monitoring. For example, our approach first obtains a new data set and subsequently analyses it for collisions; we can only detect collisions once every movement cycle, whereas tactile sensors could detect collisions at any given time. A movement cycle is defined as a discrete movement from one permanent magnet to another. This might have an impact on the reactivity of robots. The disadvantage of specialised sensory is its component cost and corresponding electronics.

## 6. Conclusion

In this study, we analysed 15 swarm robotic systems for their sensing capabilities using the instrument model from the sensing and measurement community. We exemplarily show how the measurements from HoverBot’s single Hall-effect sensor can be associated to successful movement, rotation and collision events. We constrain dynamic time warping (DTW) and DTW Barycenter Averaging (DBA) to perform time-series classification on a low-cost microcontroller. These signal processing techniques are generally applicable to time-variant data, however, must be applied to time varying, distinct, and systematically reoccurring measurements to augment a robot’s sensing capabilities. We train a classifier offline, transfer its parameters to HoverBot for online classification, and achieve high detection rates. This work shines light on how swarm roboticists can augment sensors by applying computationally constrained signal processing techniques to gain multi-functional sensing capabilities.

## Author Contributions

MN created the system and is lead author of the work. RM worked on the development of the classifier and helped writing section 3. MS worked on the introduction literature and the HoverBot system. GF worked on the development of the classifier. AH advised generally on the work. EO advised on developing the classifier. AS was the lead advisor and primary editor of the manuscript.

## Conflict of Interest Statement

The authors declare that the research was conducted in the absence of any commercial or financial relationships that could be construed as a potential conflict of interest.

## Appendix

### Sensing Modalities

We believe that some sensing modalities are more predestined for time series classification than others. In general, we can discriminate between proprioceptive and exteroceptive sensing capabilities. Both terms owe their origins to biology; the former one describes the sense of stimuli that are produced and perceived within an organism; the latter one describes the sense of stimuli that are external to an organism. For a better understanding, we created Table 3 containing all sensors that were used by swarm robotic systems listed in Table 1 and categorised them into proprio- and exteroceptive sensors.

### Artificial Signatures

Since exteroceptive sensors provide information about the environment, we can modify the environment to influence measurements. We call signatures that are manually *created* rather than naturally occurring, *artificial signatures*. This work here presents artificial signatures; we embedded permanent magnets into the arena surface to create collision, successful movement, and rotation signatures. Rather than trying to think about the measurands that we wanted to sense with HoverBot’s Hall-effect sensor, we had to ask ourselves what its measurements look like over time at several occasions.

### Natural Signatures

Proprioceptive sensors provide information about a robot’s internal state. Popular examples are actuator, orientation or movement feedback (e.g., wheel encoder, IMU, or accelerometer). Proprioceptive sensor readouts are mostly independent of environments and therefore platform independent. Since environmental modifications have little or no effect on proprioceptive sensor readings, we infer that we cannot influence their measurements either. We call signatures built from proprioceptive measurements *natural signatures.* Proprioceptive measurements are less easy to manipulate than exteroceptive measurements. Of course, there are exceptions such as detecting collisions with an accelerometer; there might be environmental modifications that could lead to different signatures e.g., by embedding springs into obstacles to influence their mechanical response during collision.

### Augmenting Existing Sensors

Artificial signatures, such that we built, are very specific to the robot system and not easily transferable, however, one can become creative and make their own environmental modifications and create signatures to exploit a robot’s existing sensing capabilities. Finding or *creating* signatures requires a thorough understanding of the system at hand. Please find below a couple of examples.

An accelerometer seems to be an ideal candidate for time sequence classification. Acceleration continuously changes over time with robot movement. An accelerometer is a proprioceptive sensor; therefore, signatures occur naturally. Now, we have to take a look at the acceleration measurements during specific events and investigate whether we can build signatures that suffice the three criteria: (1) contain time varying measurements (2) are systematically reoccurring and (3) are distinct to other signatures. Intuitively, the acceleration measurements should look different for collisions and successful movements. The acceleration measurements should also change in magnitude and frequency for movements on increasingly rough surfaces potentially allowing terrain detections. We postulate that the accelerometer does not suffer from low signal-to-noise ratios.

*Physical measurement variable:* “acceleration”

*Measurands:* “collisions”, “successful movements”, “terrain”

A time of flight (TOF) ranging sensor determines the distance to objects by measuring the time it takes a light beam to travel to an object and back. You can implement a TOF sensor pointing upwards to measure the distance to objects above the robot. Since TOF sensors are exteroceptive sensors, we have to think about environmental modifications that help creating signatures. For example, we could install a 3D surface above the robot. The surface could have periodically reoccurring patterns similar to our magnetic fields illustrated in Figure 4 depicting a “reference map” at any given time. Any *straight* movement should result in a periodically reoccurring measurement pattern which could potentially allow to verify a movement, collision and distance itself. While this setup is more difficult to establish than using the accelerometer, it uses a completely new dimension, the area above a robot, that could allow a robot to perform a variety of new tasks. The surfaces can be arbitrarily changed; each surface being a new robot environment with its own challenges. For example, a robot swarm could search for objects in the “sky” investigating meta-heuristic search strategies. The interested reader is referred to Senanayake et al.’s literature review on search strategies for swarm robots (Senanayake et al., 2016).

*Physical measurement variable:* “light”

*Measurands:* “distance”, “collisions”, “successful movements”

**Table 3:** List of all sensors from the swarm robotic systems in Table 1. Sensors are divided into proprioceptive and exteroceptive sensors.

**Proprioceptive Sensors**	**Exteroceptive Sensors**
Wheel Encoder	IR Transceiver
Torque Sensor	Humidity Sensor
Accelerometer	Tactile Sensor
Inclinometer	Microphone
Gyroscope	Temperature Sensor
Voltagemeter	Light Sensor
	Camera
	Radio
	Antenna
	Force Sensor
	RFID Reader
	Magnetometer
