# Supplementary material for: Multi-Functional Sensing for Swarm Robots Using Time Sequence Classification: HoverBot, an Example
Source: Front Robot AI. 2018 May 17;5:55. doi: 10.3389/frobt.2018.00055 (PMC7806055; doi:10.3389/frobt.2018.00055)
Supplement: Supplementary file 1 [file Table1.DOCX]

**Supplemental Information**

**Table S1 Caption:** Comparison of 15 swarm robotic systems’ sensing capabilities

| **Year** | **Robot Name** | **Measurand** | **Physical Measurement Variable** | **Sensor** |
| --- | --- | --- | --- | --- |
| (Mondada, Franzi, and Ienne 1994) | **Khepera** | Proximity/Distance  Ambient IR Light Intensity | Infrared Light | IR Sensor |
|  |  | Odometry | Magnetic Field | Wheel Encoder |
| (Caprari and Siegwart 2003) | **Alice** | Proximity/Distance  Local Communication | Infrared Light | IR Sensor |
| (Mondada et al. 2003) | **S-bot** | Proximity/Distance | Infrared Light | IR Sensor |
|  |  | Humidity | Humidity | Humidity Sensor |
|  |  | Strain | Force | Tactile Sensor |
|  |  | Torque | Force | Torque Sensor |
|  |  | Local Communication | Sound | Microphones |
|  |  | Temperature | Temperature | Temperature Sensor |
|  |  | Ambient Light Intensity | Visible Light | Light Sensor |
|  |  | Robot Detection  Color Detection | Visible Light | Camera |
|  |  | Movement Acceleration | Acceleration | Accelerometer |
|  |  | Tilt | Acceleration | Inclinometer |
|  |  | Odometry | Magnetic Field | Wheel Encoder |
|  |  | Global Communication | E. Magnetic Field | Radio |
| (Kornienko, Kornienko, and Levi 2005) | **Jasmine** | Proximity/Distance  Local Communication | Infrared Light | IR Sensor |
| (McLurkin et al. 2006) | **SwarmBot** | Global Communication | E. Magnetic Field | Radio |
|  |  | Collision | Force | Bump skirt |
|  |  | Ambient Light Intensity | Visible Light | Light Sensor |
|  |  | Vision | Visible Light | Camera |
|  |  | Local Communication | Infrared Light | IR sensor |
| (Turgut et al. 2007) | **Kobot** | Global Communication  Local Communication | E. Magnetic Field | Antenna (XBee) |
|  |  | Proximity | Infrared Light | IR Sensor |
| (Mondada et al. 2009) | **E-puck** | Proximity  Ambient IR Light Intensity | Infrared Light | IR Sensor |
|  |  | Inclination  Collision Detection  Free-Fall Detection  Movement Acceleration | Acceleration | Accelerometer |
|  |  | Vision  Long Range Distance | Visible light | Camera |
|  |  | Localisation | Sound | Microphones |
| (Bonani et al. 2010) | **MarXbot** | Proximity/ Distance  Bearing | Infrared Light | IR sensor |
|  |  | Odometry | Acceleration | Accelerometer + Gyroscope |
|  |  | Strain | Force | Force sensor |
|  |  | Vision  Distance | Visible Light | 2xCameras |
|  |  | Global Communication | E. Magnetic Field | Bluetooth, WiFi |
|  |  | RFID Tag Information | E. Magnetic Field | RFID Reader |
| (Rubenstein, Ahler, and Nagpal 2012) | **Kilobot** | Local Communication  Distance | Infrared Light | IR Sensor |
|  |  | Ambient Light | Visible Light | Light Sensor |
| (McLurkin et al. 2013) | **R-one** | Local Communication  Localisation | Infrared Light | IR Sensor |
|  |  | Odometry | Acceleration | Accelerometer + Gyroscope |
|  |  | Robot Position | Visible Light | Camera |
|  |  | Ambient Light | Visible Light | Light Sensor |
|  |  | Odometry | Visible Light | Wheel Encoder |
|  |  | Global Communication | E. Magnetic field | Radio |
| (Riedo et al. 2013) | **Thymio II** | Proximity | Infrared Light | IR Sensor |
|  |  | Global Communication | Infrared Light | IR Receiver (remote control) |
|  |  | Acceleration | Acceleration | Accelerometer |
|  |  | Touch | Force | Capacitive Touch Sensor |
|  |  | Temperature | Temperature | Temperature Sensor |
|  |  | Sound | Sound | Microphone |
| (Farrow et al. 2014) | **Droplet** | Distance  Bearing  Local Communication | Infrared Light | IR Sensor |
| (Pickem, Lee, and Egerstedt 2015) | **GRITSBot** | Distance  Bearing | Infrared Light | IR Sensor |
|  |  | Odometry | Acceleration | Accelerometer + Gyroscope |
|  |  | Battery Level | Voltage | Battery Voltage Sensor |
| (Wilson et al. 2016) | **Pheeno** | Odometry | Magnetic Field | Wheel Encoder |
|  |  | Heading | Magnetic Field | Magnetometer |
|  |  | Movement Acceleration  Odometry | Acceleration | Accelerometer |
|  |  | Proximity | Infrared Light | IR Sensor |
|  |  | Object Identification | Visible Light | Camera |
|  |  | Global Communication | E. Magnetic Field | WiFi |
| (Nemitz et al. 2017) | **HoverBot** | Global Communication | Infrared Light | IR sensor |
|  |  | Odometry  Collision Detection  Rotation Detection | Magnetic Field | Hall-effect Sensor |
